# Realization of large transmitted optical Goos–Hänchen shifts in photonic crystal slabs

**DOI:** 10.1515/nanoph-2022-0387

**Published:** 2022-09-19

**Authors:** Shihao Du, Wenjie Zhang, Wenzhe Liu, Yanbin Zhang, Maoxiong Zhao, Lei Shi

**Affiliations:** State Key Laboratory of Surface Physics, Key Laboratory of Micro- and Nano-Photonic Structures (Ministry of Education) and Department of Physics, Fudan University, Shanghai 200433, China; Department of Physics, The Hong Kong University of Science and Technology, Clear Water Bay, Kowloon, Hong Kong, China

**Keywords:** Goos–Hänchen shift, photonic crystal, transmission phase

## Abstract

In this letter, we demonstrate the existence of large transmitted optical positive and negative Goos–Hänchen shifts in photonic crystal slabs numerically and experimentally. The Goos–Hänchen shift we observed directly reaches ∼20 μm, which is 30 times the wavelength, approaching the radius of the beam waist. To explain this phenomenon, we measure the transmission phase through the phase measurement system and find there is an acute phase change near the resonance in photonic crystal slabs, which enhances the Goos–Hänchen shift. Our experimental results are consistent with simulation, and the work can be used to modulate the propagation of light in further research.

## 1 Introduction

A light beam will undergo a lateral shift in the plane of incidence when it launches onto the interface between two media, different from Newton’s corpuscular theory of optics. This lateral shift, called Goos–Hänchen (GH) shift, was first observed experimentally by Goos and Hänchen in 1947 [1]. The GH shift has attracted much attention due to its underlying physics and potential applications in many fields like optical switches [2], polarization beam splitters [3], optical sensors [4], and optical information storage [5]. In 1948, the stationary phase method was proposed by Artmann to explain the GH shift in theoretical analysis by using the phase difference between incident light and reflected light [6] and proved that the GH shift is directly proportional to the partial derivative of the reflection phase to its incident angle. It is also suitable for transmitted light [7]:

(1) $$GHS=-\frac{1}{k\cos\left(\theta \right)}\frac{\partial \phi_{t,r}}{\partial \theta }=-\frac{\partial \phi_{t,r}}{\partial k_{x}}$$

Equation (1) shows that the GH shift can be positive and negative depending on the partial derivative. The GH shift based on the sign can be divided into two categories: positive GH shift and negative GH shift. The negative GH shift as a novel counterintuitive phenomenon has attracted much attention from the beginning [7–9].

In the experiment of Goos and Hänchen, the GH shift in total internal reflection is almost in the same order of magnitude as the wavelength, much smaller than the size of the beam waist, which poses a challenge in the experimental measurement and limits its application enormously. Therefore, lots of approaches have been proposed to enhance the GH shift, including cladding waveguides [10], graphene-based structures [11, 12], gradient metasurface [13], and optical bound states in the continuum [14–16]. In these works, the largest GH shift achieved by previous subwavelength structural methods reaches approximately 0.4 times the beam waist radius [17].

Photonic crystal (PhC) slabs, due to designable band structures and complex features in the momentum space, are very significant in the fields of modulating light [18–20]. There would be a series of Bloch resonant modes, which can be classified as radiative modes within the light cone and nonradiative modes out of the light cone. These radiative modes can be coupled by plane waves in free space, which we call the guided resonance modes. Fano resonance occurs when the guided resonance modes of PhC slabs are coupled with the incident light [21, 22]. Fano resonances arise from the interaction of spectrally narrow discrete states with a continuous spectrum. Fano resonances may appear in many situations, such as the guided mode resonances (GMR) [23] and near optical bound states in the continuum [24]. Moreover, there would typically be a sharp phase change in Fano resonance, which could be used to enhance the GH shift [15, 16]. Soboleva et al. observe the GH shift at photonic crystal surfaces by weak measurement [25]. Wong et al. realize a large reflected optical Goos–Hänchen shift in photonic crystal mirror of low-loss material [9], which is about 0.1 times the beam waist radius. Wu et al. [16] realize giant GH shift theoretically in the compound structure composed of a four-part periodic grating layer and a waveguide layer close to about 0.6 times the waist radius of the beam.

In this letter, we design one-dimensional (1D) photonic crystal slabs to enlarge the optical positive and negative transmitted GH shift. We use the finite-difference time-domain method to calculate the transmittance spectra. Moreover, the relationship between transmission phase and incident angle and the specific displacement with the Gaussian beam incidence are gained by simulation. In the experiment, we directly measure the phase distribution in momentum space and observe both large positive and negative transmitted optical GH shifts, which are ∼30 times the wavelength at most and reach up to the radius of the beam waist.

## 2 Simulations and discussion

**Figure 1:**
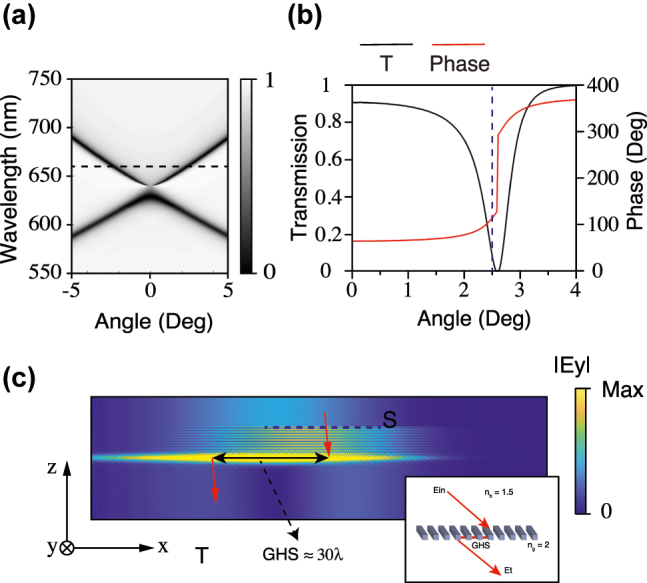
Simulated results: (a) the transmittance spectra of the grating. (b) Transmission coefficient of the grating and phase of transmitted field at *λ* = 660 nm. (c) The simulated electric field distributions of the GH shift. The inset at the bottom-right corner indicates the transmitted optical Goos–Hänchen shift diagram.

Here, we choose a 1D PhC slab (the grating) to enhance GH shift, as shown in the illustration embedded inside Figure 1(c). The grating’s refractive index is 2, corresponding to Si_3_N_4_, while the background refractive index is set to 1.5, corresponding to PMMA. The grating thickness is chosen to be 150 nm, and the period of the etched array is 400 nm. The duty cycle of the grating is 0.5. Figure 1(a) shows the calculated angle-resolved TE transmittance spectra of the sample, performed by the finite-difference time-domain method. There are valleys in the transmission spectra. They strongly depend on the incident angle, exhibiting the dispersion of the grating. The dispersion determines the grating’s responses to the external light fields. The incident light can couple with the mode on the dispersion and excite resonances. There will be a phase change around resonance, which can be used to enlarge the GH shift. We use the incident light at *λ* = 660 nm as an example. The black dotted line represents the wavelength in Figure 1(a).

The angle-resolved transmittance spectra with coefficient and phase at *λ* = 660 nm are shown in Figure 1(b). In the range of resonance, the transmitted beam has a drastic phase change, which can be used to enhance GH shift. The transmitted beam undergoes a phase change at the incident angle from 2.1° to 2.9°. The partial derivative of the transmission phase is the largest at angle *θ* = 2.6°. The transmitted GH shift is proportional to the partial derivative of the transmission phase to the incident angle, as shown in Equation (1). Therefore, the GH shift will reach the maximum when the partial derivative is the maximum and decreases as the incident angle is far from the resonance angle. Theoretically, the displacement can reach ∼50 times the wavelength with a plane wave incidence. However, the transmission coefficient is 0 at *θ* = 2.6°, as an example. Furthermore, the width of the plane wave is infinite, which means the displacement cannot be resolved. In practice, we generally apply a Gaussian beam. The beam’s incident angle is set to 2.5°, where the partial derivative of the transmission phase is near maximum, and the transmission coefficient is not 0. The blue dotted line represents the angle in Figure 1(b). Note that the partial derivative of the transmission phase is positive, so the GH shift should be negative according to Equation (1).

Figure 1(c) shows the simulated electric field distributions of the GH shift, performed by the finite-difference time-domain method. A Gaussian beam of 660 nm is obliquely shined at the sample at incident angle *θ* = 2.5°. The waist radius of the Gaussian beam is 20 μm. The GH shift of the Gaussian beam incident is the average of the transmission coefficient multiplied by the Gaussian wave packet multiplied by the gradient within the resonance angle range [26]:

(2) $$GHS=\frac{1}{T}\int G\left(k_{x}^{\prime }\right)\cdot t\left(k_{x}^{\prime }\right)\cdot \left(-\frac{\partial \phi_{t}^{\prime }}{\partial k_{x}^{\prime }}\right)dk_{x}^{\prime }$$

where *G* is the amplitude of the Gaussian wave packet, *t* is transmission coefficient, $\phi_{t}^{\prime }$ is transmission phase, and *T* is normalized constant. The GH shift can reach ∼30 times the wavelength under the incident Gauss beam in theory. The red arrows represent the central axes of the incident and transmitted beams. The dotted blue line S marks the location of the incident beam. It can be seen that part of the transmitted beam is not shifted in Figure 1(c), because the transmittance of the on-resonance band is low. One can see that the simulated transmitted GH shift reaches ∼30 times the wavelength approaching the beam waist radius, so the GH shift can be distinguished easily. Moreover, the direction of the GH shift is opposite to the direction of the incident angle. The GH shift is negative, consistent with the phenomenon in Figure 1(b).

## 3 Experimental methods and results

**Figure 2:**
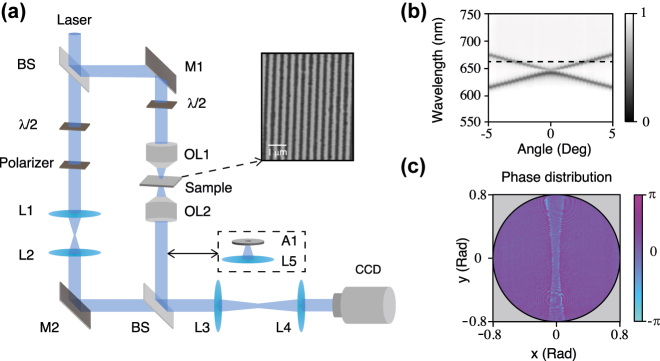
Experimental system and results. (a) Schematic diagram of the interferometric imaging phase measurement system and experimental setup for measuring the transmitted optical GH shift when A1 and L5 are placed in the light path: BS1, beam splitter; L1, L2, L3, and L4, lenses; OL1, objective lens; OL2, achromatic doublet lens. A1, mounted Pinhole, 200 μm. The inset at the right is an SEM image of the grating. (b) The measured angle-resolved transmittance spectra of the grating under TE-polarized incidence beam. (c) Measured phase distributions with TE-polarized incidence beam at *λ* = 660 nm induced by the sample.

In the experiment, we fabricated the designed grating by etching a ribbon array out from Si_3_N_4_ (*n* = 2) thin film on a SiO_2_ (*n* = 1.5) substrate with the help of electron-beam lithography and reactive-ion etching technique. PMMA (*n* = 1.5) was spun onto the grating to embed the grating in a medium (*n* = 1.5). The parameters of the sample are the same as designed. A scanning electron microscopy image of the grating is given in the inset of Figure 2(a). The overall dimensions of the fabricated sample are approximately 100 μm × 100 μm, whereas the total number of unit cells is 250. We applied our home-made Fourier-optics-based momentum space imaging spectroscopy system [27] to obtain the angle-resolved transmittance spectra. The results are shown in Figure 2(b) under TE-polarized incidence beam. The measured angle-resolved transmittance spectra agree well with our simulation results. In the following GH experiment, we set the wavelength of the incident light at *λ* = 660 nm (represented by the black dotted line in Figure 2(b)), and the incident angle is set to 2.5°, corresponding to the wavelength.

In order to measure the transmission phase of the sample, we developed the interferometric imaging phase measurement system [28]. A schematic of the experimental setup of the system is shown in Figure 2(a). The laser beam is split by a beam splitter (BS) into an object beam and a reference beam. In the optical path of the object beam, we use an objective and one achromatic doublet lens to image the beam on the charge-coupled device (CCD). The reference beam is incident on the CCD obliquely to realize off-axis interference in the optical path of the reference beam. In addition, a halfwave plate (*λ*/2) was placed in the interferometric imaging phase measurement system to analyze the responses of the incident beam with TE and TM polarization states.

Measured phase distributions induced by the resonance of the sample at a wavelength of 660 nm are illustrated in Figure 2(c). It can be seen from the measured phase distributions that there is an acute phase change from −π to π near the resonance angle as expected. Moreover, the partial derivative of the transmission phase is positive along the positive *x*-axis, which agrees with the simulation results. According to Equation (1), a positive gradient phase change will generate a negative GH shift. It will be seen in the followings.

Figure 2(a) also illustrates a schematic diagram of the experimental setup for measuring the transmitted optical GH shift. In the GH experiment, the optical path of the reference light is moved out, and A1 and L5 are placed in the object light path. The mounted pinhole (A1) was placed on the Fourier blade plane. The mounted pinhole filters the light in momentum space. The transmitted light around 2.5° is allowed to pass through the mounted pinhole. After L5, L3, and L4, the intensity distribution of the outgoing beam is captured by CCD to observe the beam directly.

**Figure 3:**
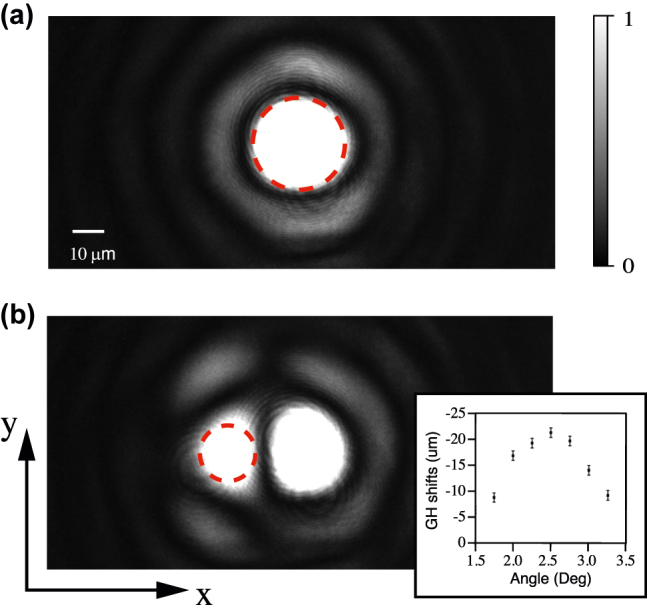
The intensity distribution of the transmitted light beam measured experimentally. (a) Transmitted beam image with TE polarized at 660 nm passing through unstructured Si_3_N_4_ window. The dashed red circle is the region of interest. (b) Beam image passing through the fabricated gratings. The other conditions are the same in (a). The inset at the bottom-right corner indicates transmitted optical GH shift angular spectra.

Figure 3(a) and (b) shows the intensity distribution of the transmitted light beam without and with the grating. The dashed red circle marks the beam that produces displacement. The transmitted beam is shifted in the negative direction compared to the beam without the grating, which means the GH shift we gained is negative. The transverse beam shift is ∼20 μm, which reaches the size of the beam waist radius. So the GH shift can be directly observed in the experiment. Figure 3(b) inset gives the transmitted optical GH shift angular spectra at *λ* = 660 nm. It is seen that the measured GH shift reaches the maximum value of 20 μm at resonance angle *θ* = 2.5° and decreases as the angle of incidence move away from the resonance angle.

**Figure 4:**
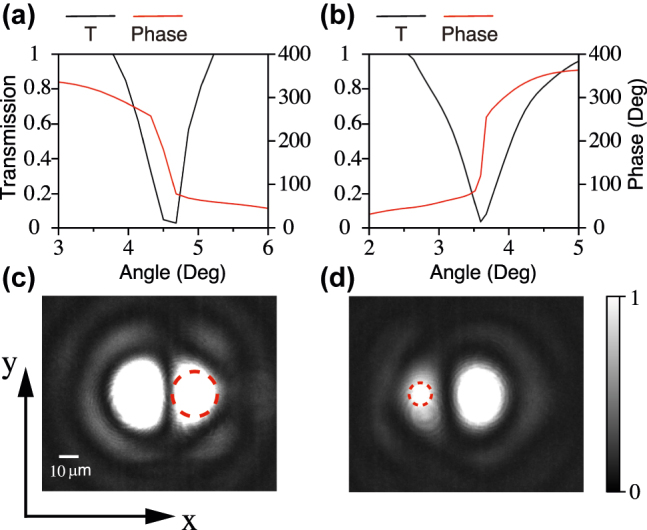
Transmission coefficient of the grating and phase of the transmitted field under TE-polarized incidence light at the wavelength (a) *λ* = 750.8 nm and (b) *λ* = 811 nm. Beam images passing through the grating at the wavelength (c) *λ* = 750.8 nm and (d) *λ* = 811 nm.

The transmitted optical GH shifts are generated by the resonance of the PhC slabs. The phase changes of different dispersion of the photonic crystal slabs are different, so the positive and negative shifts can be realized simultaneously. In order to achieve the enlarged positive and negative shifts simultaneously under our laboratory conditions, we fabricated a new grating. The period of the etched array is 500 nm. The duty cycle of the grating is 0.4. The transmittance angular spectra with coefficient and phase at *λ* = 750.8 nm and *λ* = 811 nm are shown in Figure 4(a) and (b). In the range of resonance angle, the transmitted beam has a negative transmission phase change at *λ* = 750.8 nm. While the transmitted beam has a positive transmission phase change at *λ* = 811 nm. The intensity of the transmitted beam passing through the grating was measured as shown in Figure 4(c). There is a positive GH shift at *λ* = 750.8 nm, reaching ∼20 μm. The intensity of the transmitted beam passing through the grating at *λ* = 811 nm was measured as shown in Figure 4(d). The GH shift at *λ* = 811 nm is negative. Note that the grating we designed is only considered for the incident light of TE polarization. Under the same conditions, there is no dispersion for the incident light with TM polarization. Hence, there will be no GH shift either.

The efficiency of the transmitted GH shift is low due to the acute change in the phase near the transmission dip. Wu et al. [15, 16] and Zheng et al. [29] realize enhanced GH shift with near-unity reflectance by utilizing quasi-bound states in the continuum. In the grating system, a similar situation exists, as shown in Figure S2, and the reflected GH shift is shown in Figure S3(a). In order to further enhance the GH shift, we notice that it is a bound state in the continuum protected by symmetry at the Γ point, as shown in Figure 1(a). Figure S4 shows that the GH shift becomes more significant as the wavelength gets closer to the Γ point. At the same time, the angular range of the resonance region becomes smaller. The divergence angle of the incident Gaussian beam should be smaller for the resonance. The beam waist radius of the Gaussian beam should be larger correspondingly. Moreover, the coupling efficiency may reduce. Considering the above factors and our laboratory conditions, we chose the incident wavelength as 660 nm. The proposed large transmitted optical Goos–Hänchen shifts in photonic crystal slabs can improve the functionality of sensitive sensors [30, 31] even though their efficiency is low. And the significant shifts may affect the further development of light information storage devices [32] and optical switches [33].

## 4 Conclusions

In conclusion, we studied the transmitted optical GH shift in photonic crystal slabs theoretically and experimentally. Since photonic crystal slabs can interact with the optical field resulting in an acute phase change, the transmitted GH shifts can be enhanced using photonic crystal slabs. We observed the transmitted optical GH shift experimentally, and with different dispersion of photonic crystal slabs, both positive and negative shifts can be achieved. The GH shift in the grating system can be utilized to design optical sensors, light absorption, and optical switches.

## Supplementary Material

Supplementary Material Details
