# Supplementary material for: Realization of large transmitted optical Goos–Hänchen shifts in photonic crystal slabs
Source: Nanophotonics. 2022 Sep 19;11(20):4531–6. doi: 10.1515/nanoph-2022-0387 (PMC11501337; doi:10.1515/nanoph-2022-0387)
Supplement: Supplementary file 1 — Supplementary Material Details [file j_nanoph-2022-0387_suppl_001.pdf]

Realization of large transmitted optical Goos–Hänchen shifts in photonic crystal slabs: Supplementary Information

Shihao Du[1], Wenjie Zhang[1], Wenzhe Liu[2], Yanbin Zhang[1], Maoxiong Zhao[1], and Lei Shi[1,*]

[1] State Key Laboratory of Surface Physics, Key Laboratory of Micro- and Nano-Photonic Structures (Ministry of Education) and Department of Physics, Fudan University, Shanghai 200433, China

[2] Department of Physics, The Hong Kong University of Science and Technology, Clear Water Bay, Kowloon, Hong Kong, China

* Corresponding author: lshi@fudan.edu.cn

S1. Transmitted optical GH shift angular spectra

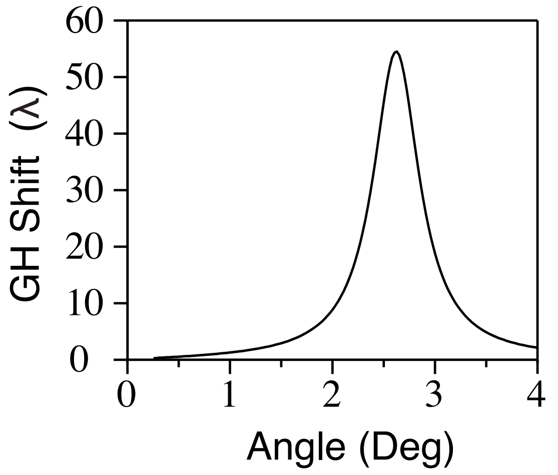

Fig. S1: Transmitted optical GH shift angular spectra under the plane wave incidence. The GH shift reaches the maximum, ~50 times the wavelength, at $\theta = 2.6°$.

S2. Reflectance and reflectance phase

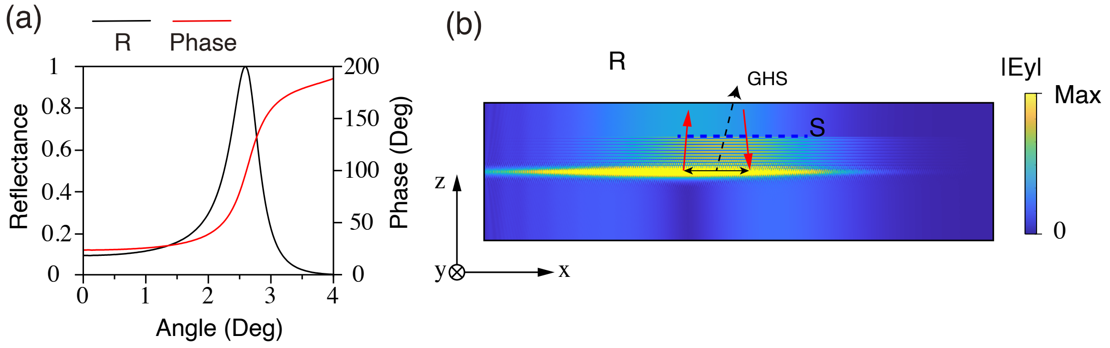

Fig. S2: (a) The reflectance of the grating and phase of the reflected field at λ = 660 nm. The reflectance reaches near-unity at $\theta = 2.6°$. It can be seen in the proposed grating system that the reflection phase also changes around the resonance angle. Hence, the reflected GH shift can also be enlarged. (b) The simulated electric field distributions of the GH shift under the Gaussian beam incidence of the grating. The red arrows represent the central axes of the incident and reflected beams.

S3. The simulated electric field distributions of the GH shift under the Gaussian beam incidence

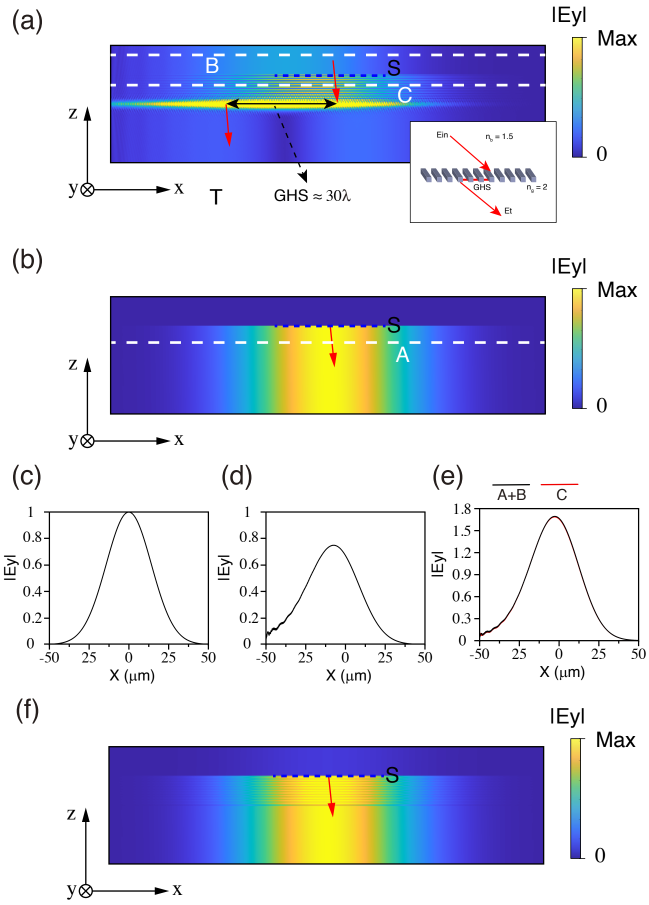

Fig. S3: (a) The simulated electric field distributions of the GH shift under the Gaussian beam incidence of the grating. Compared to Figure 1(c), we adjusted the original colorbar to observe the electric field in the interference region. The solid black line S marks the location of the incident light. The white dotted line A identifies the electric field position in the interference region. The white dotted line B identifies the electric field position of the reflection region. The blue arrow represents the reflected beam. (b) The simulated electric field distributions of the GH shift under the Gaussian beam incidence without the grating. The white dotted line c identifies the position of the incident optical field. (c) The electric field distribution of the incident light is identified by the white dotted line C. (d) The electric field distribution of the reflected light is identified by the white dotted line B. (e) The solid red line marks the electric field distribution in the interference region marked by the white dotted line C. The solid black line marks the sum of the electric field distributions of (c) and (d). It can be seen that the solid red line and the solid black line almost overlap. Hence, the interference light is produced by the superposition of the original incident light and reflected light. (f) The simulated electric field distributions with a thin film (n=2).

S4. Transmission of the grating and phase of transmitted field and transmitted optical GH shift angular spectra

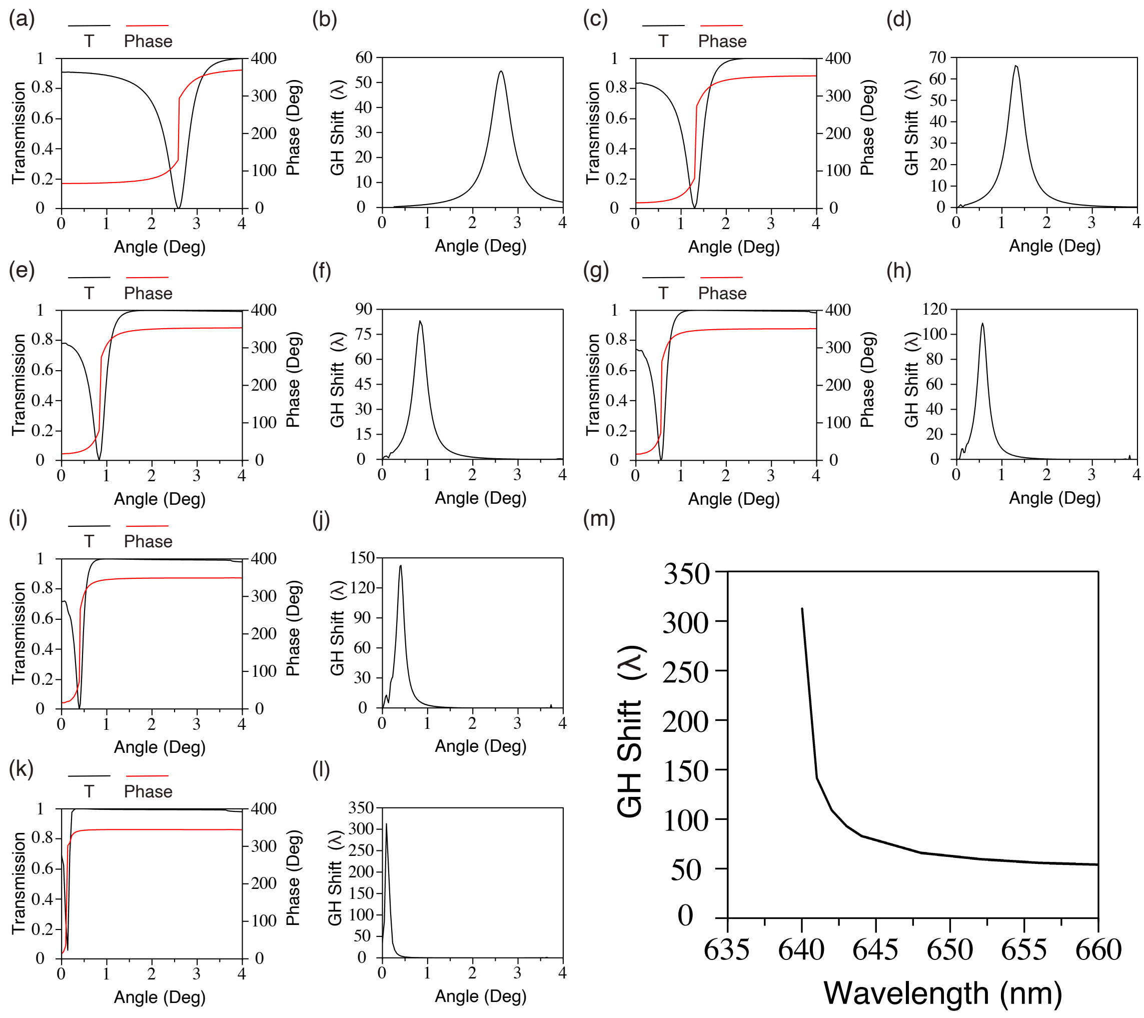

Fig. S4: Transmission of the grating and phase of transmitted field and transmitted optical GH shift angular spectra of the grating under the plane wave incidence at λ = 660 nm (a)(b), at λ = 648 nm (c)(d), at λ = 644 nm (e)(f), at λ = 642 nm (g)(h), at λ = 641 nm (i)(j), at λ = 640 nm (k)(l). (m) The relationship between the transmitted optical GH shift and the incident wavelength.

S5. Transmitted GH shifts

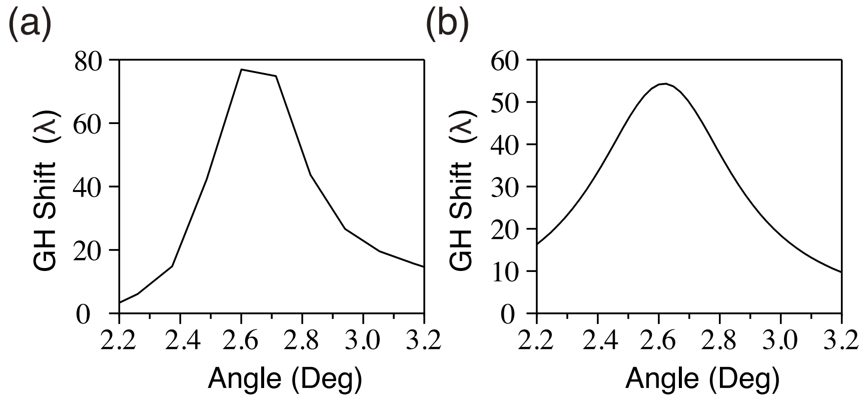

Fig. S5: (a) Transmitted optical GH shift angular spectra obtained from the experimental transmission phase angular spectra in the region of phase change around 0 along the x-axis in Fig. 2c. (b) Transmitted optical GH shift angular spectra obtained by simulation.

S6. SEM images of the grating

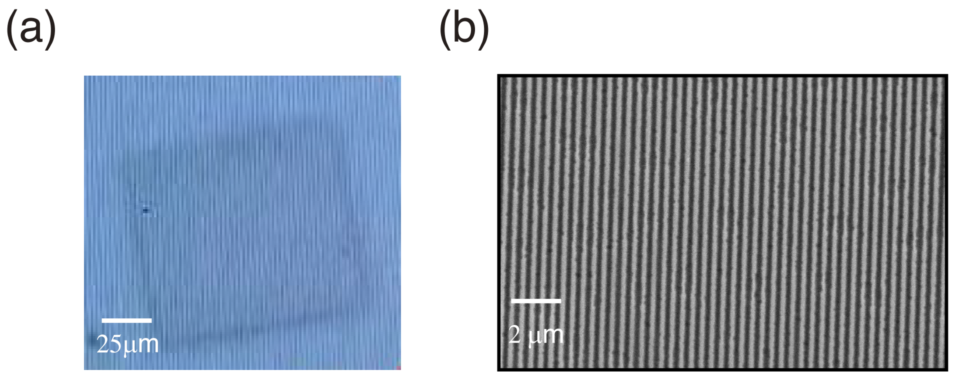

Fig. S6: (a) An SEM image of the grating. The overall dimensions of the fabricated samples are approximately 100 μm × 100 μm. The number of repeating structures is 250. The stripes in the blue area originate from the computer screen and are fake. (b) Partially enlarged view of (a).
